# Construction and Analysis of Coexpression Network to Understand Biological Responses in Chickens Infected by *Eimeria tenella*

**DOI:** 10.3389/fvets.2021.688684

**Published:** 2021-07-09

**Authors:** Baohong Liu, Xueting Ma, Jianping Cai

**Affiliations:** ^1^State Key Laboratory of Veterinary Etiological Biology, Key Laboratory of Veterinary Parasitology of Gansu Province, Lanzhou Veterinary Research Institute, Chinese Academy of Agricultural Sciences, Lanzhou, China; ^2^Jiangsu Co-innovation Center for Prevention and Control of Important Animal Infectious Diseases and Zoonoses, Yangzhou, China

**Keywords:** weighted gene coexpression network analysis, *Eimeria tenella*, infection, modules, immune response

## Abstract

Coccidiosis, caused by various *Eimeria* species, is a major parasitic disease in chickens. Our understanding of how chickens respond to coccidian infections is highly limited at both the molecular and cellular levels. In this study, coexpression modules were identified by weighted gene coexpression network analysis in chickens infected with *Eimeria tenella*. A total of 15 correlation modules were identified using 5,175 genes with 24 chicken samples, 12 with primary and 12 with secondary *E. tenella* infection. The analysis of the interactions between these modules showed a high degree of scale independence. Gene Ontology and Kyoto Encyclopedia of Gene and Genomes enrichment analyses revealed that genes in these functional modules were involved in a broad categories of functions, such as immune response, amino acid metabolism, cellular responses to lipids, sterol biosynthetic processes, and RNA transport. Two modules viz yellow and magenta were identified significantly associating with infection status. Preservation analysis showed that most of the modules identified in *E. tenella* infections were highly or moderately preserved in chickens infected with either *Eimeria acervulina* or *Eimeria maxima*. These analyses outline a biological responses landscape for chickens infected by *E. tenella*, and also indicates that infections with these three *Eimeria* species elicit similar biological responses in chickens at the system level. These findings provide new clues and ideas for investigating the relationship between parasites and host, and the control of parasitic diseases.

## Introduction

Chickens are an important component of agricultural economy worldwide, as they serve as one of the primary sources of protein for humans. However, *Eimeria* infections inflict significant economic losses on the poultry industry, as they cause the host with decreased nutrient absorption, retarded growth, reduced egg production, and mortality (1, 2). The genus *Eimeria* includes seven species (*Eimeria acervulina, Eimeria maxima, Eimeria tenella, Eimeria mitis, Eimeria necatrix, Eimeria praecox*, and *Eimeria brunetti*), each with distinct avian coccidiosis pathogenicity and immunogenicity (3, 4). *Eimeria acervulina, E. maxima*, and *E. tenella* are the most common species infecting commercial poultry (5). Cornelissen et al. compared immune responses to infections with a single *Eimeria* species and with a mixture of *E. acervulina, E. maxima*, or *E. tenella* and found that the strongest immune response was induced in the specific part of the intestine affected by each *Eimeria* strain (5). Kim et al. compared the transcriptomes of the three species in chickens with primary and secondary infection and found that *E. tenella* elicited the most gene alterations in both primary and secondary infection, while few genes were differently expressed in primary infection and many genes were altered in secondary infection with *E. acervulina* and *E. maxima*. Pathway analysis demonstrated that the altered genes were involved in certain intracellular signaling pathways. All their analyses were based on differentially expressed genes (DEGs) or single cytokines that were identified as isolates (6). Although differential expression studies have provided insights into the pathogenesis of *Eimeria*, discovering that gene associations using the system biology approach will deeply improve our understanding at the mechanistic and regulatory levels. Weighted gene coexpression network analysis (WGCNA) is a technique for identifying gene modules within a network based on correlations between gene pairs (7, 8), which has been used to study genetically complex diseases (9–11) as well as agricultural sciences (12–15). In this study, we constructed the weighted gene coexpression network (WGCN) on the microarray datasets of chickens infected by *E. tenella*, delineated the module functions, and examined the module preservation across *E. acervulina* or *E. maxima* infection, which is aiming to reveal the biological responses elicited by *E. tenella* infection and the conserved responses among chickens infected with different *Eimeria* species at a system level and shedding light on the mechanisms underlying the infection's progression.

## Materials and Methods

### Microarray Harvesting and Processing

**Figure 1:**
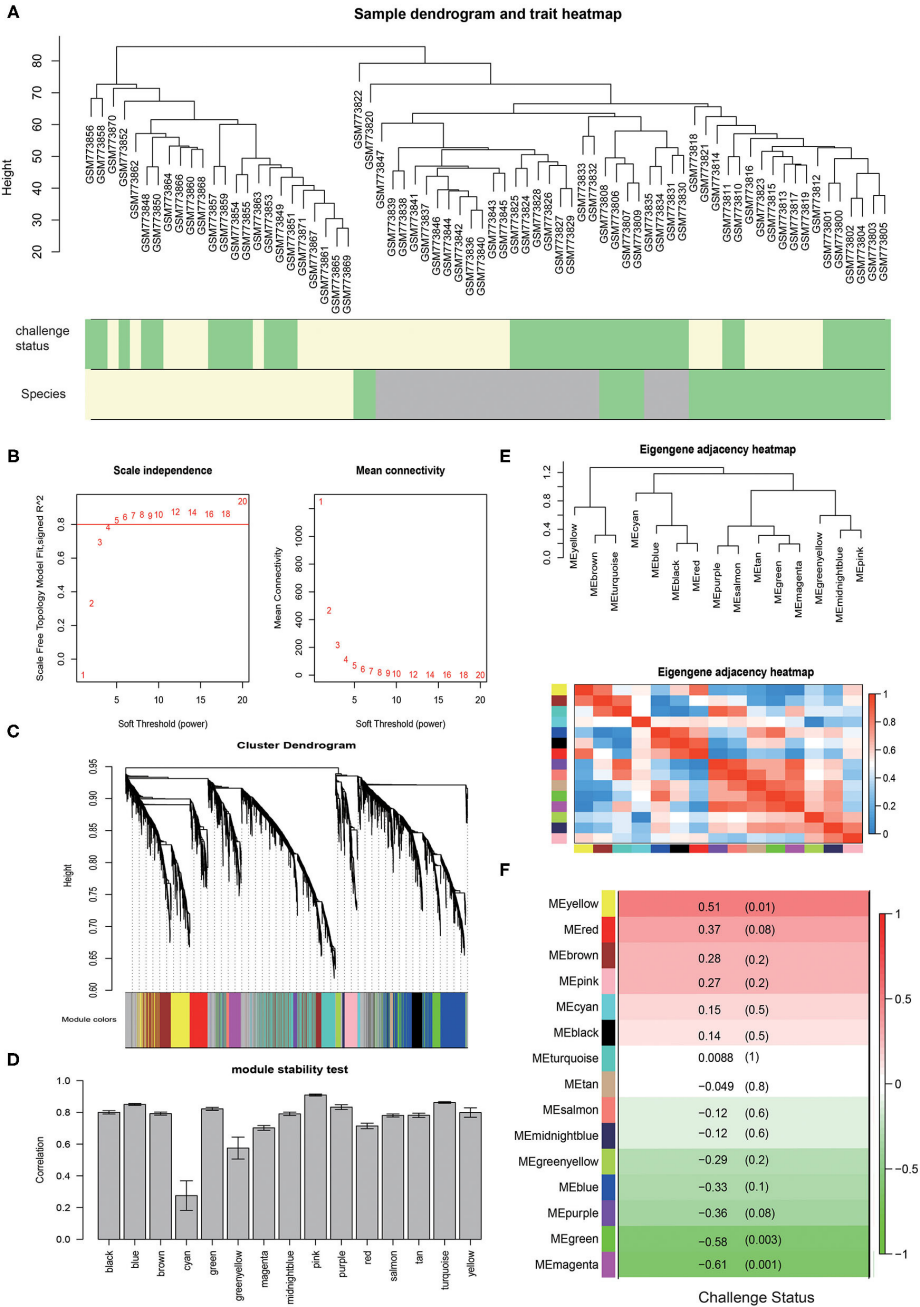
The WGCNA results for chickens infected with *E. tenella*. **(A)** The samples clustering for chickens infected by *E. acervulina* (lightgreen), *E. maxima* (gray), and *E. tenella* (lightyellow) with the primary infection (lightgreen) and secondary infection (lightyellow). **(B)** The scale independence curve and the mean connectivity curve. **(C)** The dendrogram for the modules constructed by WGCNA. **(D)** Correlation of intramodule connectivity for each module after sampling 1,000 times (mean ± sd). **(E)** Module clustering and heatmap. **(F)** The module-trait analysis results.

The expression dataset was downloaded from the database of Gene Expression Omnibus (GEO) (https://www.ncbi.nlm.nih.gov/geo/) with the accession number of GSE31213 (Figure 1A). This data set was derived from microarray analysis of chicken intestinal intraepithelial lymphocytes between one and 6 days post-primary and secondary infection with *E. acervulina, E. maxima*, and *E. tenella* (6). Uninfected control samples and one of the three infection group samples were labeled with different fluorescent dyes and hybridized simultaneously on the same slide using a reference design with a dye swap protocol. Consequently, there were 24 samples per species, including 12 samples with primary and 12 with secondary infection. As there are 21,168 probe sets, we streamlined the dataset by excluding probe sets with no GenBank accession number and combining probe sets with same numbers, thus obtaining probe sets with unique GenBank accession number. We then downloaded the sequences from the National Center for Biotechnology Information (NCBI) according to the GenBank accession number and BLAST of the chicken genome with an e value <e−10, and obtained 7,671 probe sets. For the gene with multiple probe sets, we retained the probe set which was most often associated with the highest expression level across samples (16). Finally, 5,175 genes were achieved. The dataset was quantile normalized using the “normalizeQuantiles” function of the *R* package limma (17).

### Construction of a Weighted Gene Coexpression Network

WGCNA method was applied to calculate the appropriate power value which was used to construct the weighted network (7). The suitable power value was determined when the degree of scale independence was set to 0.8 using a gradient test. The coexpression modules (clusters of interacted genes) were constructed by the function of “blockwiseModules” using the above power value. Then, the genes in each corresponding module was obtained. For the reliability of the result, the minimum number of genes in each module was set to 30. Cytoscape (v3.7.1) was used to visualize the coexpression network of module genes (18).

To test the reproducibility of the identified modules, a sampling test was performed by the in-house *R* script, in which half of the samples (six primary infection samples and six secondary infection samples) were randomly selected to calculate the new intra module connectivity. The sampling was repeated 1,000 times and then the module stability was represented by the correlation of intra module connectivity between the original and the sampled ones (19).

### Gene Ontology and KEGG Pathway Enrichment for Each Coexpression Module Gene List

Gene Ontology (GO) enrichment and Kyoto Encyclopedia of Gene and Genomes (KEGG) pathway analyses for each interacted module were performed using *R* package of clusterProfiler (20). The 5,175 genes remaining after the pre-process were set as the enrichment background, and *p*-value <0.05 was the significance criteria.

### Module-Trait Relationships

To select potentially biologically interesting modules for downstream analysis, Spearman's correlation between the module eigengene and infection traits (infection status viz primary vs. secondary infection) was calculated. The eigengene is the first principal component of a given module and a representative measure of gene expression profile in the module.

### Module Preservation Analysis

Our module preservation analysis was based on a permutation test performed using the *R* “modulePreservation” function (7), which includes several powerful network-based statistics. These statistics are summarized in the composite preservation called Zsummary. For each module in the reference data set of *E. tenella* infected chickens, the function calculates the Zsummary statistic in the test data set of *E. acervulina* or *E. maxima* infected chickens. For a given module, a Zsummary value of >10 indicates strong evidence for preservation in the test data set, whereas a value of <2 indicates no evidence.

## Results

### Construction of Coexpression Modules of Chickens Infected With *E. tenella*

The expression values of the 5,175 genes in chickens infected with *E. tenella* were used for the construction of the reference coexpression modules by the WGCNA package. We set the power value to 5 according to the scale independence curve and the mean connectivity curve (Figure 1B). Finally, a total of 15 coexpression modules were constructed (Figure 1C). A total of 860 genes, accounting for 16.62%, were not assigned to any of these modules. We assigned a color to and counted the number of genes in each module. There were 863 genes in the turquoise module, 788 in the blue module, 635 in the brown module, 432 in the yellow module, 382 in the green module, 270 in the red module, 205 in the black module, 192 in the pink module, 179 in the magenta module, 134 in the purple module, 76 in the green-yellow module, 47 in the tan module, 42 in the salmon module, 37 in the cyan module, and 33 in the midnight blue module.

### Module Stability Test

The module stability showed that module pink, turquoise, blue, purple, green and black were among the most stable modules (connectivity correlation >0.8). Module cyan displayed the least stability (Figure 1D).

### Analysis of the Coexpression Module Interactions

We analyzed the relationships between the 15 coexpression modules. Module eigengenes in this analysis were defined as the first principal component of a coexpression module matrix. Cluster analysis was performed on these eigengenes (Figure 1E). The connectivity degree of eigengenes was determined to better understand the interactions between the coexpression modules. The heatmap in Figure 1E showed the relatedness of the 15 coexpression modules identified by WGCNA, with red indicating close relatedness and blue indicating no relatedness. The results demonstrated that the gene expression of each module was mutually exclusive, indicating a high degree of scale independence.

### Coexpression Modules Significantly Correlated With Different Infection Status

To identify modules related to primary and secondary infection, we calculated the correlations between module eigengenes and the infection status (Figure 1F). The modules were selected using a correlation *p*-value of <0.05 as a threshold. The genes in the magenta (*R* = −0.61, *p* = 0.001) and yellow (*R* = 0.51, *p* = 0.01) modules are significantly positively or negatively correlated to the infection status.

### Coexpression Module Function Enrichment

**Figure 2:**
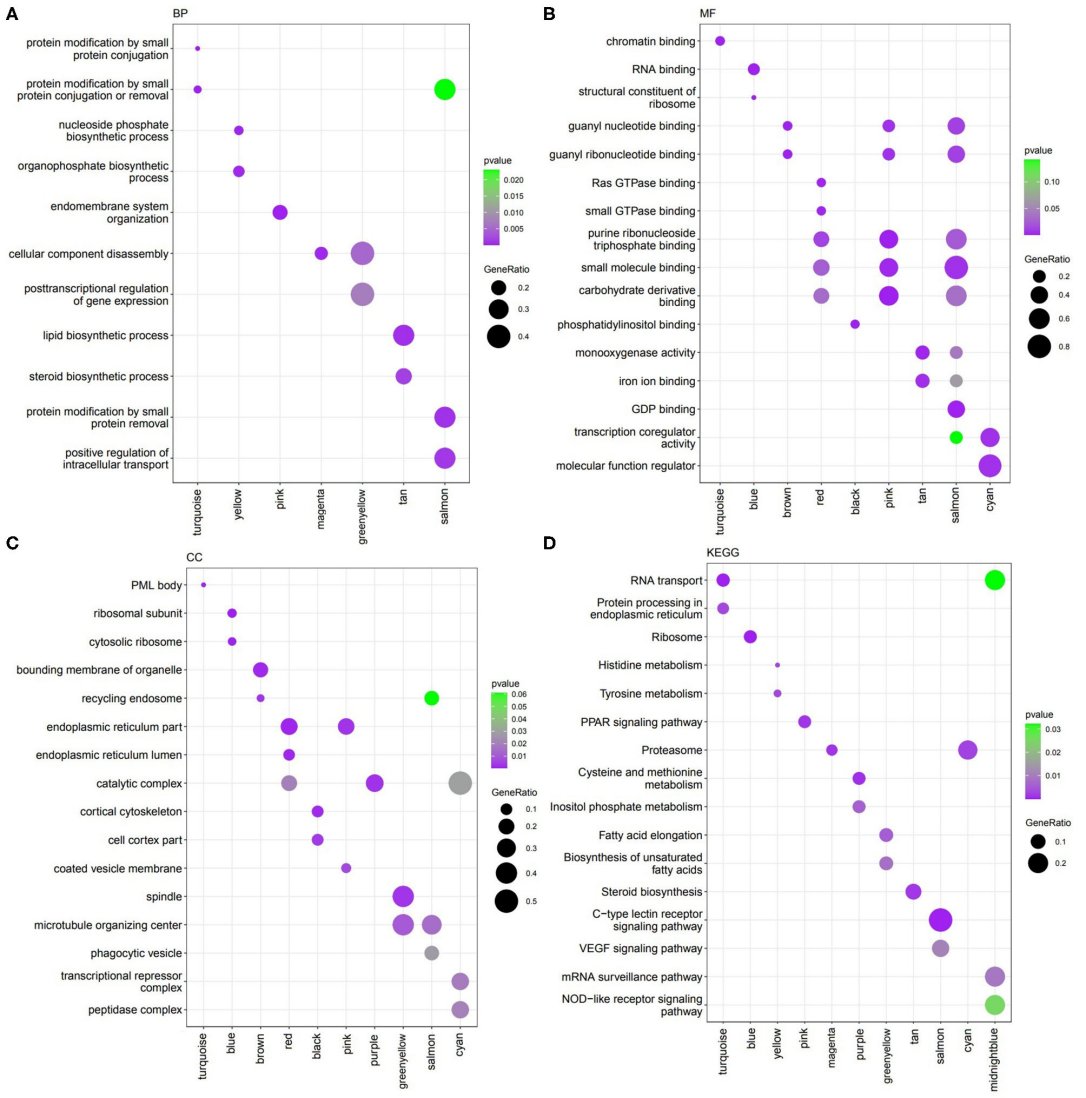
Functions identified by clusterProfiler. **(A)** Biological Process (BP). **(B)** Molecular Function (MF). **(C)** Cellular Component (CC). **(D)** KEGG pathways.

The Biological significance of modules was conducted by GO and KEGG pathway enrichment analaysis. The results revealed that all the modules significantly enriched at least one or more GO terms or pathways (Figure 2 and Table 1). The biological functions of the modules were broadly catagorized into metabolism, transport, gene expression, repair, immune response, lipid biosynthesis, and protein metabolism and modification.

**Table 1:** Gene ontology (GO) and KEGG pathway functional annotation of genes in the gene coexpression network.

**Module**	**Term ID**	**Term/Keg**	**nGenes**	**Percentage**	***p*-value**	***p*.adjust**	**Term name**
Turquoise	gga03013	KEGG	23	0.079	7.26E-06	9.95E-04	RNA transport
	GO:0032446	BP	14	0.115	3.79E-05	4.54E-02	Protein modification by small protein conjugation
Blue	gga03010	KEGG	23	0.076	4.72E-07	6.55E-05	Ribosome
	GO:0001816	BP	9	0.078	5.00E-03	2.94E-01	Cytokine production
Brown	GO:0031400	BP	11	0.116	2.00E-03	6.13E-01	Cellular homeostasis
	GO:0008152	BP	17	0.179	1.00E-02	8.90E-01	Cell cycle
Yellow	gga00360	KEGG	3	0.019	6.50E-03	1.63E-01	Phenylalanine metabolism
	gga00380	KEGG	5	0.019	5.00E-03	1.59E-01	Tryptophan metabolism
	gga00982	KEGG	5	0.019	3.90E-03	1.59E-01	Drug metabolism - cytochrome P450
	gga00340	KEGG	4	0.019	2.80E-03	1.59E-01	Histidine metabolism
	gga00350	KEGG	5	0.019	3.00E-03	1.59E-01	Tyrosine metabolism
	GO:1901293	BP	7	0.132	2.20E-04	1.05E-01	Nucleoside phosphate biosynthetic process
Green	GO:0034976	BP	4	0.082	6.90E-04	4.63E-01	Response to endoplasmic reticulum stress
	GO:0033554	BP	12	0.245	4.00E+03	4.69E-01	Cellular response to stress
Red	GO:0097190	BP	4	0.129	4.50E-03	6.17E-01	Apoptotic signaling pathway
	GO:2001233	BP	3	0.097	6.50E-03	6.17E-01	Regulation of apoptotic signaling pathway
Black	GO:0008104	BP	9	0.310	2.40E-03	3.81E-01	Protein localization
	GO:0097435	BP	6	0.207	2.50E-03	3.81E-01	Supramolecular fiber organization
Pink	GO:0010256	BP	5	0.200	5.21E-05	3.94E-02	Endomembrane system organization
	GO:0009966	BP	9	0.360	4.58E-03	5.16E-01	Regulation of signal transduction
	gga03320	KEGG	5	0.075	1.40E-03	9.32E-02	PPAR signaling pathway
Magenta	GO:0022411	BP	5	0.172	4.00E-04	2.33E-01	Cellular component disassembly
	GO:0006955	BP	6	0.207	3.00E-03	4.39E-01	Immune response
	GO:0006952	BP	6	0.207	5.58E-03	4.39E-01	Defense response
	gga03050	KEGG	4	0.058	1.35E-03	9.87E-02	Proteasome
Purple	gga00270	KEGG	4	0.075	9.86E-04	7.39E-02	Cysteine and methionine metabolism
	GO:0007269	BP	2	0.074	8.57E-03	6.45E-01	Neurotransmitter secretion
	GO:0033077	BP	2	0.074	8.57E-03	6.45E-01	T cell differentiation in thymus
Greenyellow	GO:0010608	BP	2	0.400	7.22E-03	2.26E-01	Post-transcriptional regulation of gene expression
	GO:0051301	BP	2	0.400	1.75E-02	2.26E-01	cell division
	GO:1903047	BP	2	0.400	1.54E-02	2.26E-01	mitotic cell cycle process
	gga00062	KEGG	2	0.087	5.59E-03	1.20E-01	Fatty acid elongation
	gga01040	KEGG	2	0.087	7.99E-03	1.20E-01	Biosynthesis of unsaturated fatty acids
	gga04114	KEGG	3	0.130	9.74E-03	1.20E-01	Oocyte meiosis
Tan	GO:0008610	BP	3	0.333	5.34E-05	2.01E-01	Lipid biosynthetic process
	GO:0006694	BP	2	0.222	5.09E-04	2.01E-01	Steroid biosynthetic process
	GO:0016125	BP	2	0.222	9.49E-04	2.01E-01	Sterol metabolic process
	GO:0006629	BP	3	0.333	4.00E-03	2.89E-01	Lipid metabolic process
	gga00100	KEGG	2	0.118	1.58E-03	3.32E-02	Steroid biosynthesis
Salmon	GO:0070646	BP	2	0.333	8.44E-04	1.85E-01	Protein modification by small protein removal
	GO:0000070	BP	2	0.333	1.61E-03	1.85E-01	Mitotic sister chromatid segregation
	gga04370	KEGG	2	0.143	1.08E-02	1.35E-01	VEGF signaling pathway
Cyan	GO:0000079	BP	1	0.250	1.94E-02	2.91E-01	Regulation of cyclin-dependent protein serine/threonine kinase activity
	GO:0044774	BP	1	0.250	1.94E-02	2.91E-01	Mitotic DNA integrity checkpoint
	GO:1901989	BP	1	0.250	1.94E-02	2.91E-01	Positive regulation of cell cycle phase transition
	gga03050	KEGG	2	0.182	2.61E-03	7.05E-02	Protease
Midnight blue	GO:0000380	BP	1	0.143	3.37E-02	4.52E-01	Alternative mRNA splicing, *via* spliceosome
	GO:0045739	BP	1	0.143	3.37E-02	4.52E-01	Positive regulation of DNA repair
	gga03015	KEGG	2	0.200	9.07E-03	1.99E-01	mRNA surveillance pathway

Module turquoise involved in protein modification and RNA transport. Module yellow engaged in phosphate biosynthetic process, histidine, and tyrosine metabolism. Module pink involved in endomembrane system organization and PPAR signaling pathway. Genes in module greenyellow involved in post-transcriptional regulation of gene expression, cell division, fatty acid elongation and cell cycle. Module tan contained genes with functions of lipid and steroid biosynthesis. Salmon module involved in protein modification and C-type lectin receptor signaling pathway. Besides, blue module contained genes related to ribosome. Genes in midnight blue engaged in mRNA surveillance pathway and positive regulation of DNA repair.

### Infection Status Associated Modules Analysis

**Figure 3:**
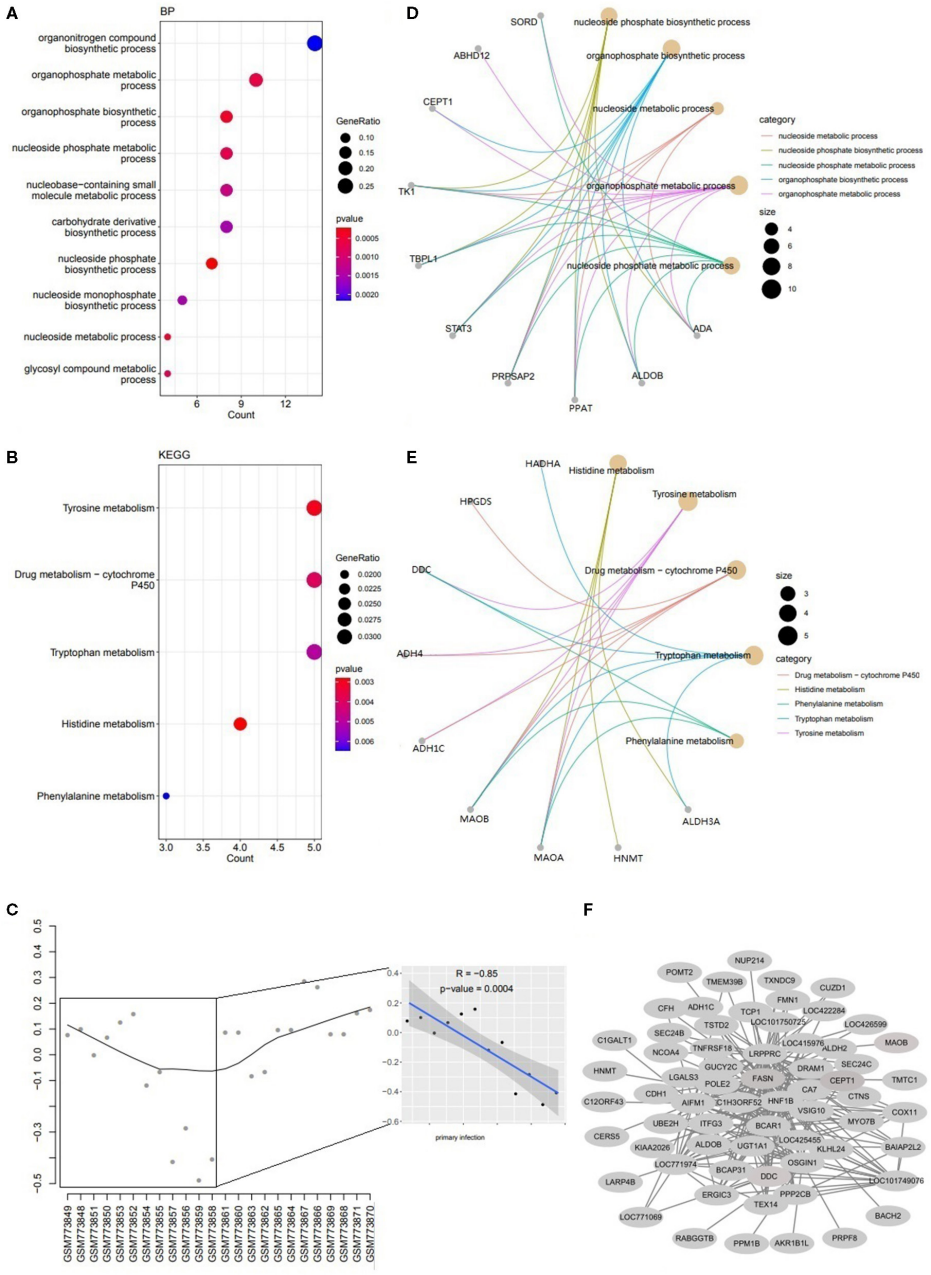
The Functions for genes in yellow module. **(A)** The dotplot of top 10 BPs. **(B)** The concept network of top 5 BPs. **(C)** The dotplot of top 5 KEGG pathways. **(D)** The concept network of top 5 KEGG pathways. **(E)** The trajectory curve and linear regression for the eigengene. **(F)** The coexpression network for module yellow.

Two modules of yellow and magenta were significantly associated with the infection status (primary vs. secondary infection) by the module-trait analysis (Figure 1E). For module yellow, genes were involved in biosynthetic and metabolism process (nucleoside phosphate, organophosphate, and carbohydrate derivative) (Figure 3A) and metabolism pathways (histidine, tyrosine, drug metabolism-cytochrome P450, tryptophan and phenylalanine) (Figure 3B). The expression level of Genes in yellow module decreased significantly in the primary infection over time and increased in the secondary infection over time (Figure 3C). The concept networks showed the details of genes in the top 5 GO terms and KEGG pathways (Figures 3D,E). The coexpression network for module yellow genes was shown in Figure 3F.

**Figure 4:**
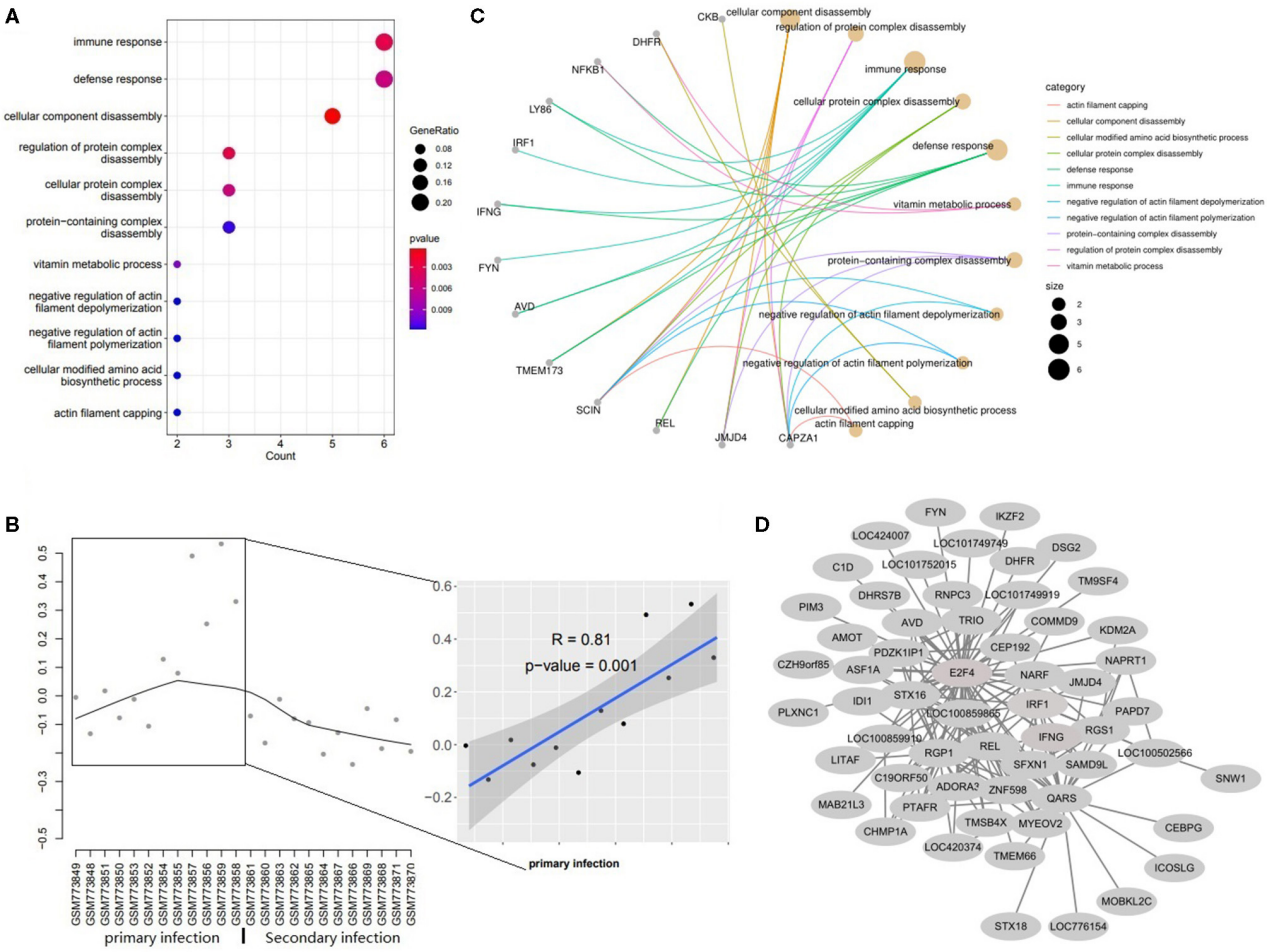
The Functions for genes in magenta module. **(A)** The dotplot of top 11 BPs. **(B)** The concept network of top 11 BPs. **(C)** The trajectory curve and linear regression for the eigengene. **(D)** The coexpression network for module magenta.

Genes in module magenta were involved in immune response, defense response and actin filaments related functions (Figure 4A). The expression level of genes in this module increased significantly in the primary infection over time (Figure 4B). The concept networks showed the details of genes in the top 11 GO terms and IRF1, IFNG, and CAPZA1 were circled as the important genes (Figure 4C) and also identified as hub genes in this module (Figure 4D).

### Most Modules Were Preserved in Chickens Infected With *E. maxima* or *E. acervulina*

**Figure 5:**
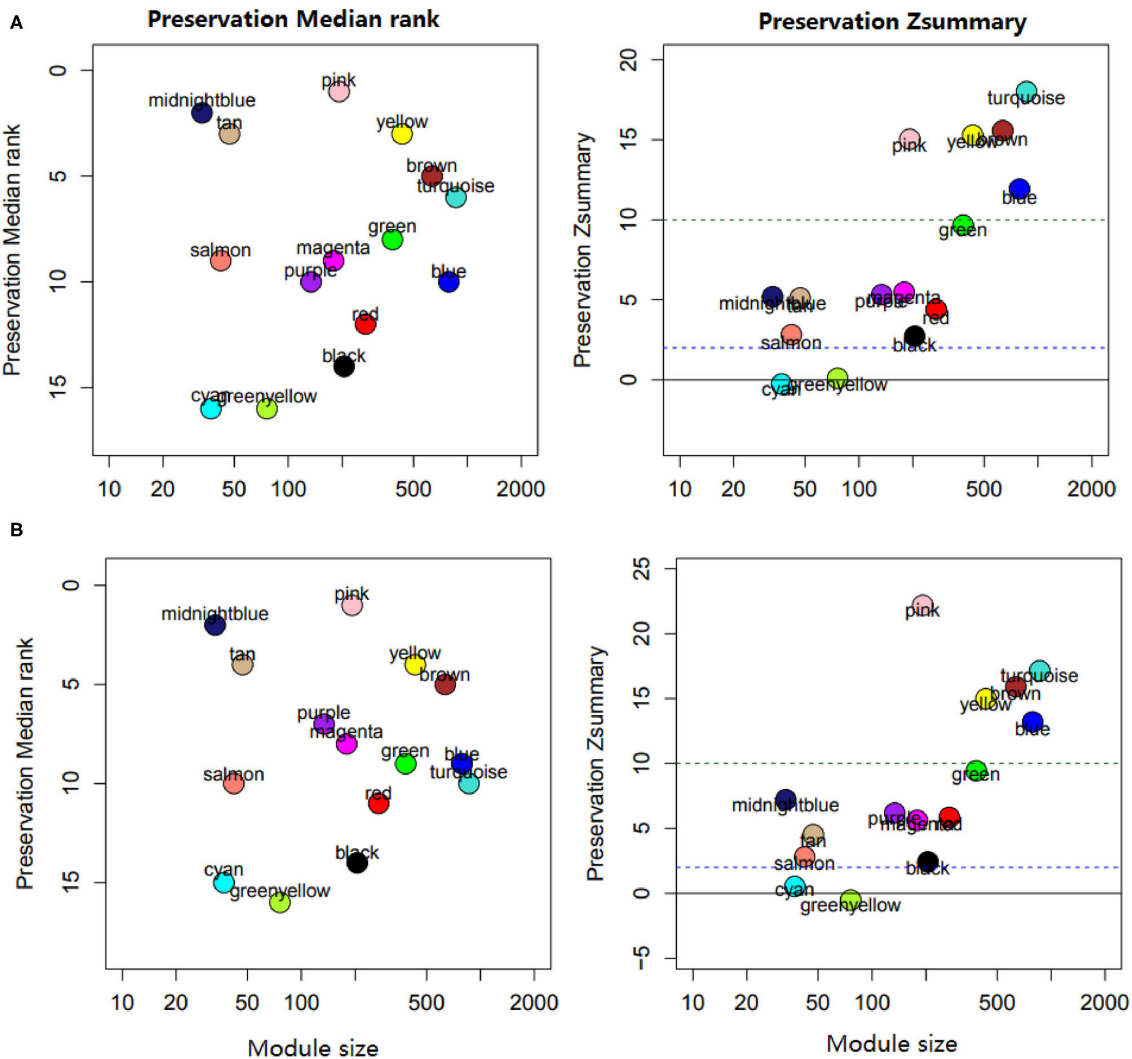
Module preservation analysis. **(A)** Modules preserved in *E. acervulina*. **(B)** Modules preserved in *E. maxima*.

Preservation analysis showed that most modules appeared in the datasets of chickens infected with *E. maxima* or *E. acervuline*, ranging from moderate to high preservation. The pink, turquoise, brown, yellow, and blue modules were highly preserved, with z scores above 10. The remaining modules showed high-to-moderate conservation, with preservation scores ranging from 2 to 10, except the cyan and greenyellow modules (Figure 5).

## Discussion

In this study, we looked for the biological responses and differences for primary and secondary infection with *E. tenella* at the level of gene coexpression regulation rather than that of individual genes. Using gene expression data, we constructed a WGCN for *E. tenella* infected chickens. Instead of conventional analysis of gene expression changes, WGCNA focuses on interactions in a batch of genes, avoiding the disadvantages of treating genes independently and ignoring the molecular transcriptional networks (16). A total of 15 mutually exclusive coexpression modules were identified for chickens infected by *E. tenella*. The functional enrichment analysis revealed a wide category of functions among the modules (Table 1).

The analysis of the association between module eigengenes and external traits showed that genes in module yellow and magenta were significantly positively or negatively correlated with the infection status. The expression of genes in the yellow module was involved in amino acids metabolism process and pathways. Tryptophan, tyrosine and phenylalanine metabolism have been reported to play vital roles in the immune response regulation (21). Hamzić found that tryptophan deprivation inhibits *Toxoplasma gondii* replication and may be involved in the innate immune response to *E. maxima* infection (22, 23). It was reported that tyrosine is a precursor to dopamine, catecholamine, and melanin production, in which dopamine is a neurotransmitter involving in the regulation of the immune response (21). Phenylalanine engaged in the regulation of nitricoxide (NO) synthesis (24) indirectly, and NO plays multiple roles relating to the immune response which can regulate the cytokine production, and killing pathogens (25). In this study, the expression of genes decreased significantly with the primary infection over time (Figure 3C) which indicated that it was inhibited for the *E. tenella* by initializing the host innate immune response.

Genes in the magenta module were involved in the immune response, defense response and actin filaments de/polymerization. It is well-known that protozoan parasite infections can trigger a severe intestinal mucosal immune response (22, 23). This is in line with the upregulated expression of genes in the magenta module under the primary challenge (Figure 4B). There are some important hub genes in this module such as IFNG, IRF1, and CAPZA1. IFNG encodes a soluble cytokine that is a member of the type II interferon class. The encoded protein is secreted by cells of both the innate and adaptive immune systems. The production of IFNG is a homodimer that binds to the interferon gamma receptor and is considered as pivotal for triggering effector mechanisms against *Toxoplasma gondii, Cryptosporidium, Plasmodium*, and *Eimeria* infections (26) and can inhibit the replication of parasites (27). IRF1 is interferon regulatory factor 1 and protein encoded by this gene is a transcriptional regulator and tumor suppressor, serving as an activator of genes involved in both innate and acquired immune responses. The encoded protein activates the transcription of genes involved in the body's response to viruses and bacteria, playing a role in cell proliferation, apoptosis, immune response, and DNA damage response (28). CAPZA1 is a capping actin protein of muscle Z-line alpha subunit 1 which encodes the α subunit of F-actin capping protein (29) and regulates actin polymerization and cell motility *via* binding to the barbed ends of actin filaments (30, 31). In the present study, all these hub genes upregulated after the primary infection, which indicates that the immune responses are activated.

Using the same gene expression dataset, we examined whether the coexpression network structure of modules identified in *E. tenella* infected chickens might be preserved in *E. acervulina* or *E. maxima* infected chickens. The highly preserved modules are mainly involved in RNA transport (turquoise), ribosome (blue), cellular homeostasis (brown), regulation of signal transduction (pink) and amino acid metabolism (yellow) which were the basic functions that maintain the normal activities of cells (32). The high-to-moderate preserved modules of green, midnight blue, purple, red, magenta, tan, salmon and black were enriched in a wide category of functions. For example, genes in module green mainly enriched in response to endoplasmic reticulum stress (ERS). During infection of parasites, ERS was induced in host cells, together with initiation of unfolded protein response (UPR) which play important roles in the immune system (33). The parasites can use this reaction in various ways to promote its survival and pathogenicity, and to influence inflammation, oxidative stress, apoptosis, and immune responses of host cells (34–36). The expression level of genes in this module with the preservation scores >9 increased over primary infection time (Supplementary Figure 1), meaning that the ESR was induced by the infection regardless of *Eimeria* species. Genes in module tan were involved in cellular lipids/steroid biosynthesis and metabolism, and many hub genes (FDPS, ERLIN1, DHCR24, and PTGES3) in this module encode proteins that are the key intermediates in cholesterol and sterol biosynthesis/metabolism (37–40). Cellular cholesterol content is controlled under strict homeostasis by a feedback regulatory system (41). Intracellular pathogens evolved mechanisms to subvert host metabolism and may use host lipid bodies, as ways of immune evasion and nutrients source (42). The cholesterol content increased (Supplementary Figure 2) in the inflammatory tissues with enhanced antioxidant capacity, making them less susceptible to oxidative stress, thus conferring a selective growth advantage to pathogens (40, 42).

## Conclusion

In summary, we identified multiple coexpression gene modules with a wide category of functions in chickens infected by *E. tenella* for the first time. Two modules were screened out to be positively or negatively correlated to the infection status with functions of amino acid metabolism and immune responses indicating the difference between primary infection and secondary infection. Most of the coexpression module structures were similar for all pairwise comparisons between *E. tenella* infection and other *Eimeria* species infection. The highly preserved coexpression modules enriched genes to maintain the cell normal activities. The high-to-moderate modules include genes playing pivotal roles of responses to parasites infection. Thus, the preservation analysis indicated the infection of different *Eimeria* species can elicit similar responses at the system level. These findings provide new clues and ideas for investigating the relationship between parasites and host, and the control of parasitic diseases. Thus far, we found only one microarray dataset of *E. tenella* infected chickens that included 24 samples meeting the sample size requirement for use of the WGCNA. Still, we need to watch for more RNA-seq/microarray datasets to verify the results identified here in the future.

## Data Availability Statement

Publicly available datasets were analyzed in this study. This data can be found in online repositories. The names of the repository/repositories and accession number(s) can be found in the article/Supplementary Material.

## Author Contributions

BL contributed to the design and conception of this study, conducted computational experiments, performed and interpreted data, and drafted the manuscript. XM revised the manuscript. JC conceived this project and participated in its design, helped in interpreting the data, and drafted and revised the manuscript. All authors read and approved the final manuscript.

## Conflict of Interest

The authors declare that the research was conducted in the absence of any commercial or financial relationships that could be construed as a potential conflict of interest.
