# Supplementary figures and images for: Construction and Analysis of Coexpression Network to Understand Biological Responses in Chickens Infected by Eimeria tenella
Source: Front Vet Sci. 2021 Jul 9;8:688684. doi: 10.3389/fvets.2021.688684 (PMC8299102; doi:10.3389/fvets.2021.688684)

# MEgreen

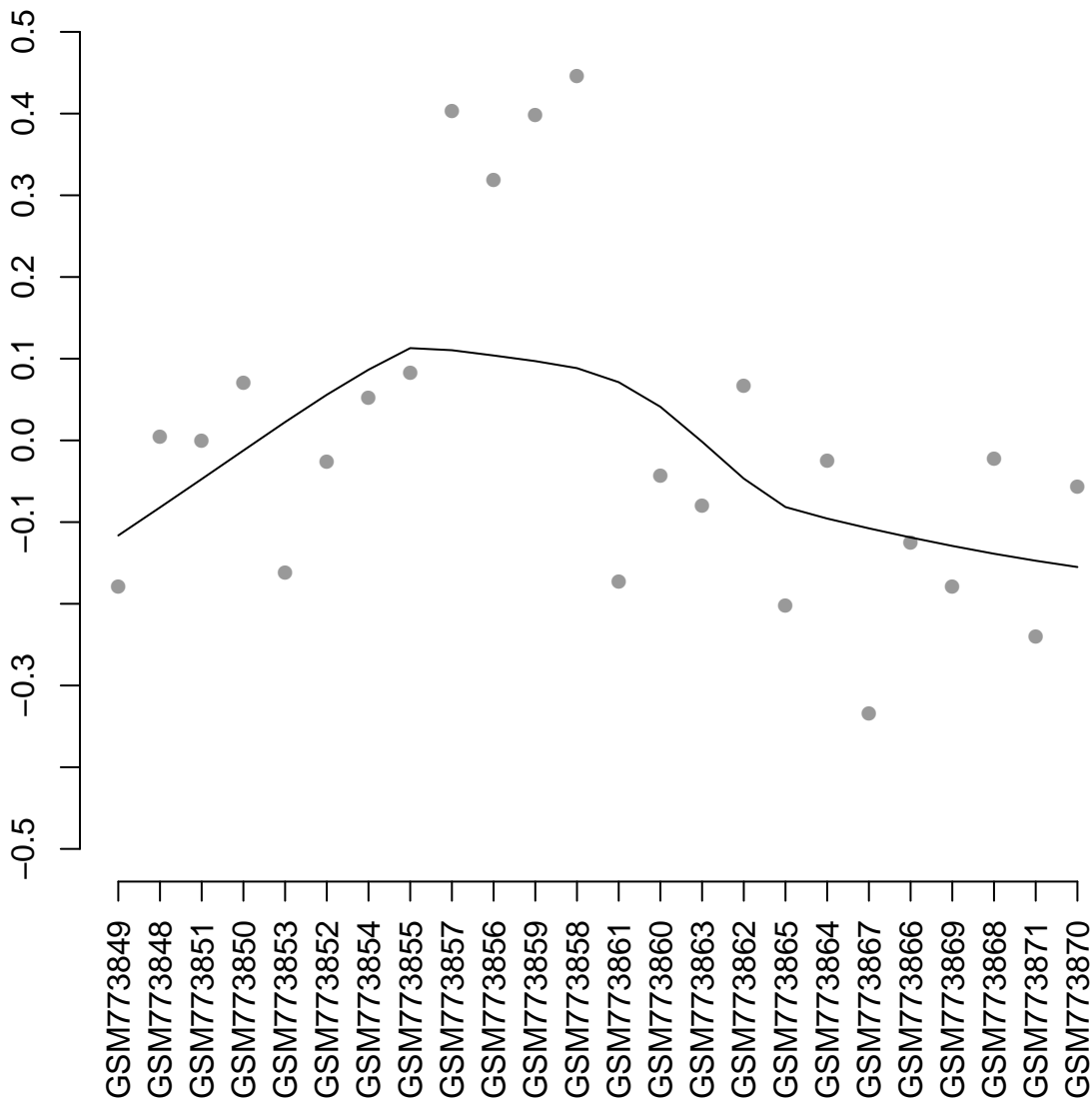

Supplement: Supplementary file 1 [file Data_Sheet_1.PDF]

# MEtan

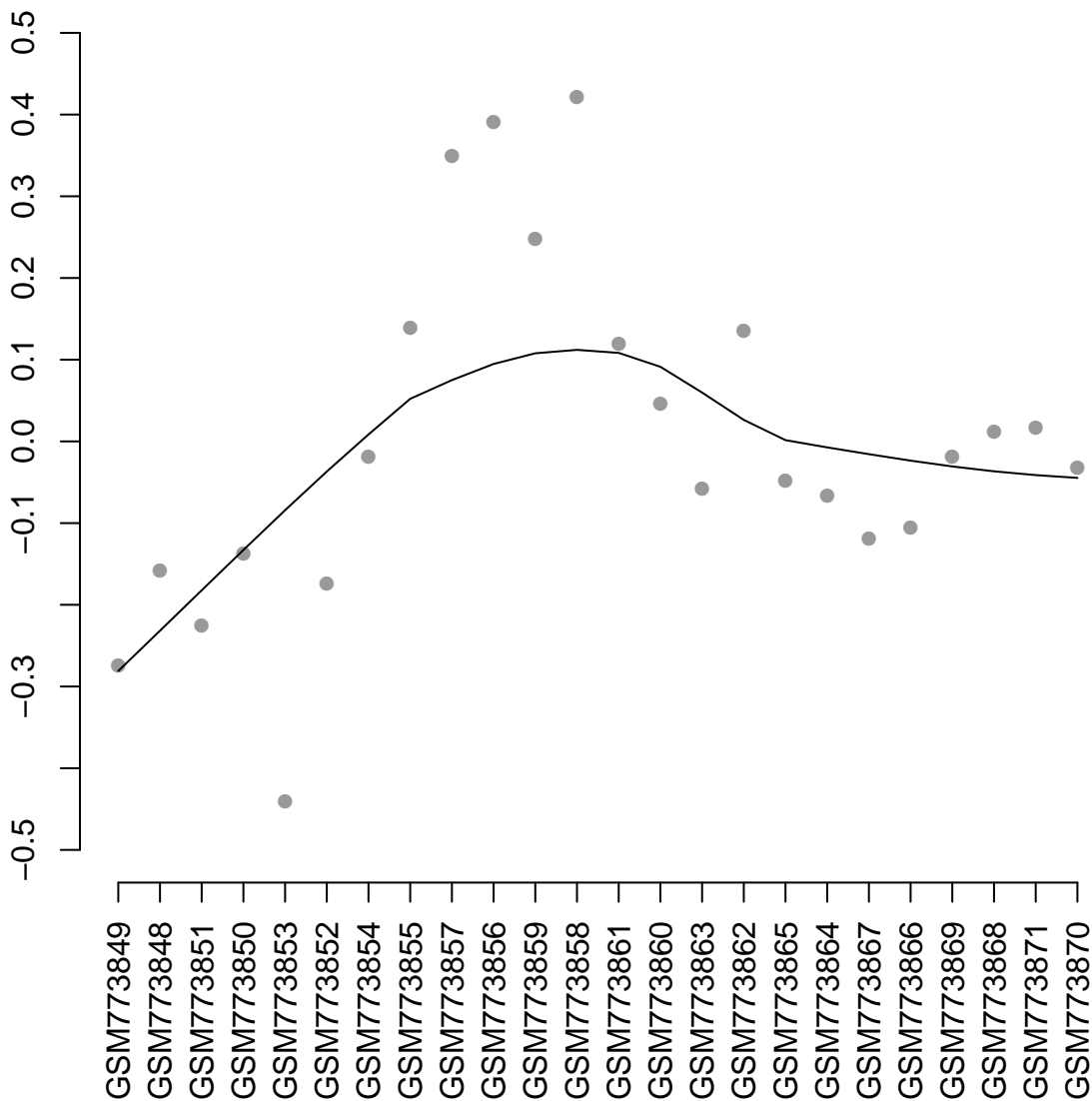

Supplement: Supplementary file 2 [file Data_Sheet_2.PDF]
